# Deficiency of MTMR14 impairs male fertility in *Mus musculus*

**DOI:** 10.1371/journal.pone.0206224

**Published:** 2018-11-09

**Authors:** Nana Wen, Meng-Fei Yu, Jing Liu, Congli Cai, Qing-Hua Liu, Jinhua Shen

**Affiliations:** 1 Hubei Provincial Key Laboratory for the Protection and Application of Special Plant Germplasm in the Wuling Area of China, College of Life Sciences, South-Central University for Nationalities, Wuhan, China; 2 Wuhan Youzhiyou Biopharmaceutical Co., Ltd., Biolake, Wuhan, China; Nanjing Medical University, CHINA

## Abstract

Calcium signalling is critical for successful fertilization. In spermatozoa, capacitation, hyperactivation of motility and acrosome reactions are all mediated by increases in intracellular Ca^2+^. Our previous reports have shown that deficiency of MTMR14, a novel phosphoinositide phosphatase, induces a muscle disorder by disrupting Ca^2+^ homeostasis. Recently, we found that MTMR14 is also expressed in the testes; however, whether deficiency of MTMR14 in the testes also alters the Ca^2+^ concentration and impairs male fertility remains entirely unknown. In the present study, we found that MTMR14 is also expressed in the testes and mature sperm cells, suggesting that deficiency of MTMR14 might have some effect on male fertility. Both *in vivo* fertility and *in vitro* fertilization tests were then performed, and we found that MTMR14^-/-^ male mice showed decreased fertility. A series of experiments were then arranged to test the testis and sperm parameters; we found that MTMR14 deficiency caused small size of the testes, small numbers of both total and immotile sperm, expanded membrane of sperm tail, a decreased proportion of acrosome reaction, and in contrast, an increased proportion of abnormal sperm and augmented apoptosis, etc. Further study also found that the muscle force of the vas deferens decreased significantly in KO mice. Intracellular calcium homeostasis in the testes and epididymis was impaired by MTMR14 deletion; moreover, the relative mRNA expression levels of *Itpr1*, *Itpr2*, and *Ryr3* were dramatically decreased in MTMR14 KO mice. Thus, MTMR14 deletion impairs male fertility by causing decreased muscle force of the vas deferens and intracellular calcium imbalance.

## Introduction

In mammals, Ca^2+^ signalling plays an important role in almost every step, such as sperm capacitation, motility and the fusion between sperm and eggs [1,2]. The intracellular Ca^2+^ concentrations can be increased by either Ca^2+^ influx through plasma membrane ion channels or Ca^2+^ release from intracellular stores; however, low intracellular Ca^2+^ concentrations are maintained through mechanisms involving the plasma membrane Ca^2+^ pump ATPase and the mitochondria [3,4]. In spermatozoa, capacitation, hyperactivated motility and acrosome reactions are all regulated by increases in intracellular Ca^2+^ concentrations ([Ca^2+^]_i_) [5].

Several Ca^2+^-permeable ion channel proteins participate in fertilization of mammalian sperms [6–10]. CatSper (a pH-regulated, calcium-selective ion channel) and KSper (Slo3) are core regulators of sperm tail calcium entry and hyperactivated sperm motility [6,7]. To date, only the four mammalian *CatSper* members (*CatSpers* 1–4) are restrictively expressed in the testes and have clearly been shown to be required for male fertility [8,9,10]. Male mice deficient in any of the four CatSpers are completely sterility but exhibit no other apparent abnormalities. KSper/Slo3, a pH-dependent K(+) current, is thought to be composed of subunits encoded by the slo3 gene, although the equivalence of KSper- and Slo3-dependent currents remains uncertain. KSper/Slo3 is the primary spermatozoon K(+) current; KSper/Slo3 may plays a pivotal role during the acquisition of normal morphology and sperm motility when sperms are faced with hyperosmotic challenges, and KSper/Slo3 is critical for fertility [11,12]. With the development of the sperm patch-clamp technique, CatSper and KSper have been confirmed as the primary spermatozoon ion channels [13]. In addition, many other channels have been proposed to play a role in regulating sperm activity without direct measurements, including the voltage-gated proton channel Hv1 in human sperm tails and the P2X2 ion channel identified in the midpiece of mouse sperm [14,15]. Mutations and deletions in sperm-specific ion channels such as L-typ Ca^2+^ channels affect male fertility in both mice and humans without affecting other physiological functions. The uniqueness of sperm ion channels makes them ideal pharmaceutical targets for contraception. Thus, it is very worthwhile to identify and characterize new proteins that might affect male fertility by regulating ion channels in sperm.

MIP/MTMR14, a novel phosphoinositide phosphatase, and its inactivating mutations were found in human centronuclear myopathy in 2006 [16,17]. Since then, its mouse and zebrafish homologies have also received much attention. To further clarify its function, Dr. Qu’s lab knocked out the gene encoding this phosphatase and characterized some of the resulting phenotypes as follows. First, they found that deficiency of this gene induces a muscle disorder by disrupting Ca^2+^ homeostasis [18]; In MTMR14 knockout mice, spontaneous Ca^2+^ leakage from the sarcoplasmic reticulum occurred. This leakage was due to the decreased metabolism/dephosphorylation and the following accumulation of MIP substrates such as PI(3,5)*P*2 and PI(3,4)*P*2. second, they found that MTMR14 might also be involved in regulation of the ageing process [19,20]; and third, they found that MTMR14 also plays an important role in the regulation of autophagy [21,22,23]. The role of MTMR14 in autophagy in zebrafish was also reported by another group [24].

It was reported that MTMR14 is highly expressed in heart and skeletal muscle, and our data showed that it is also highly expressed in the testes [18]; however, the role of MTMR14 in the testes was completely unknown. In this report, we show that deficiency of MTMR14 significantly impaired male fertility; accordingly, the total number of sperm, the ratio of motile sperm, and the proportion of sperm undergoing acrosome reaction were significantly decreased in MTMR14 KO mice, whereas the ratio of abnormal sperm was dramatically increased. Briefly, the spermatogenesis and fertility of MTMR14 KO mice are impaired.

## Materials and methods

### Animals

MTMR14^+/-^ mice (C57BL/6J background) were generously provided by Dr. Cheng-Kui Qu (Case Western Reserve University, U.S.A.). The mice at 6 and 12 weeks were maintained at a 12 h light/dark cycle with *ad libitum* access to food and water. The genotypes of both the original mice and their offspring were confirmed by PCR before further experiments. All of the methods and experimental protocols involving animals were performed in accordance with national and international guidelines and approved by our own institutional board (the Animal Care and Use Ethics Committee of South-Central University for Nationalities, **No: SCUEC-2016-SJH01**). This study was approved by this committee.

### Real-time PCR

Total-RNA isolation, single-strand cDNA synthesis and real-time PCR were performed according to the manufacturer’s instructions. The primer sequences used in this project are shown in the Table 1 [25].

**Table 1:** Sequence information for primers.

Gene	Forward	Reverse
*MTMR14*	5’-AGACCTCATTCACCGAAGCA-3’	5’-TGTCACCACTCCGAAGAACA-3’
*SIX5*	5’- CTGCAGTCTGAAGTCCCACA-3’	5’- ACCCCTAGTGCCCACACATA-3’
*Atg7*	5’-AAACAGGCCACAGAAAATGG-3’	5’-TGCGCTGACCTATCTGGAAT-3’
*Atg9*	5’- TCTGTTTGGCTTTGGCTTTT -3’	5’- CATAGAAGCAGCCAGGAAGG-3’
*SEPT4*	5’- TCAAGTTGAGGACGATGCTG-3’	5’- TCTCCCGGATTAGCTTCTCA-3’
*RAD51C*	5’- CCTCCACGGACACTAAGCAT-3’	5’- CACAGACCAGGAGTCAGCAA-3’
*RAD23B*	5’- TTTCAGGGCAGAAGAGCAGT-3’	5’-TCTGCAGGTTTGTGTCCAAG-3’
*ADAD1*	5’- TACAGGGAGCCTTGCTGAGT-3’	5’- CCTTGTGCCCAATTCAAACT-3’
*ZFP35*	5’- GGCCGGACTAGTTCAAGGAT-3’	5’- CCCCACAAGGCAGATTTTTA-3’
*TSNAXIP1*	5’-CACCATCCTCAAGACCACCT-3’	5’- TCTGCACAAATGGCTCACTC-3’
*Spag16l*	5′-AGCAAGCCAGAGACATCCAT-3′	5′-CCAGAAATCTTCCCAACAGC-3′
*Spag16s*	5′-CTCTGACACAATGAGTATGG-3′	5′-CTACAGGAAATTCTGAATCC-3′
*HMGB2*	5’- TAGCCCTGGCTGTCCTAGAT-3’	5’- TGCATAAGGCTCTGGGTTCT-3’
*HOOK1*	5’- CAGCAGACACAAGGGAGACA-3’	5’- GCCAGCTCAATCTGGCTTAC-3’
*Actin*	5'-AGAGGGAAATCGTGCGTGAC-3'	5'-CAATAGTGATGACCTGGCCGT-3'
*Itpr1*	5'-TGATTTCTTTTCTGGATGGGTG-3'	5'-TTGACTTGCTTCGGAACTCTG-3'
*Itpr2*	5'-TGAAGGACCCGACAGAATACAC-3'	5'-AGGCATCCGATGAAAGGCT-3'
*Itpr3*	5'-TCTCACCTCCGAGCACTACATT-3'	5'-CTTGTGAGGCTTGCCTACAAA-3'
*Ryr1*	5'-TCCGAGACCAACAAGAGCAAGT-3'	5'-CCAGACATACGACTCCTGACCA-3'
*Ryr2*	5'-ACGGATGCTCAGTCTCAGAGGA-3'	5'-AGTGTGACTGCCGTGCTTGG-3'
*Ryr3*	5'-GGTAAACCTGAGTTCACGACAAGC-3'	5'-CAAGCTGATTCTGGAGACAGTCAC-3'

### Immunohistochemical staining of testicular tissue sections

Immunohistochemical experiments were performed as described previously [26,27]. Briefly, freshly dissected testes from WT and MTMR14^-^/^-^ mice were fixed in 4% paraformaldehyde (PFA), dehydrated in 25% sucrose/PBS overnight, and sectioned. The sections were blocked with 10% normal goat serum and permeabilized in 0.3% Triton X-100. After blocking, the sections were incubated with anti-MTMR14 primary antibody (Abcam, Cat No. Ab102575) at 4°C overnight. After three washes with PBS, the sections were incubated with horseradish peroxidase donkey anti-rabbit antibody for 2 h. Then, the DAB method was performed to detect the signals. In the negative control group, normal rabbit serum IgG was used as a substitute for the primary antibody. Following preparation, the slides were analysed, and different images were captured.

### Immunofluorescence

Spermatogonial cells and mature sperm from different species were isolated as follows. The cauda epididymides and vas deferens of WT/MTMR14-deficient male mice were carefully excised. After removal of adipose tissue and blood vessels, tissues were transferred into 1 mL of PBS. The ductus deferens was squeezed gently with forceps to extrude sperm. The cauda was lightly minced and squeezed gently and then incubated at 37°C to allow the remaining sperm to swim out into the medium. The immunofluorescence of the spermatogonial cells and sperm was performed as described previously with some modifications [28]. Briefly, the sperm were isolated, placed on a coverslip and allowed to settle for 15 min. Adherent cells were washed with PBS and subsequently fixed with 4% PFA, permeabilized with 0.1% Triton X-100 in phosphate-buffered saline (PBS), and blocked in 3% bovine serum albumin (BSA) in PBS for 1 h. After incubation with primary antibody for 1.5 h, the samples were washed with PBS three times and incubated in diluted FITC-conjugated secondary antibody for 1 h. After counter-staining with propidium iodide (PI), the images were collected with a Zeiss LSM700 confocal microscope (Zeiss, Germany).

### Fertility testing

The fertility of the MTMR14^-/-^ males was investigated by mating with WT females. Mating tests (male:female = 1:1)were performed with 28 homozygous male mice (MTMR14^-/-^) for at least 3 months. Detailed information about the experimental design is shown in **Table 2**. Females were checked for the presence of vaginal plugs and/or pregnancy. Pregnant females were removed to holding cages to give birth. The numbers and sizes of the litters sired by each group of males were determined.

**Table 2:** MTMR14^-/-^ male mice were sub-infertile.

Matings			Number of matings	Litters	Proportion pregnant (%)	Total number of offspring	Offspring per litter (Mean±S.D.)
Group	♂	♀					
1	WT	WT	28	28	100	210	7.5±0.9
2	MTMR14^-^/^-^	WT	28	14	43	99	7.1±0.7
3	MTMR14^-^/^-^	MTMR14^-^/^-^	28	11	39	85	7.7±0.8

Note: The mating experiments lasted for 3 months. During this period, once the female was plugged or pregnant, she was removed from the mating cage to give birth, and the number of offspring was determined.

### Sperm isolation

Sperms were isolated as previously described with some modifications [29]. Briefly, the caudal epididymis was excised and dissected from the fat, blood vessels and connective tissues. Then, it was transferred into human tubal fluid to squeeze out the sperm using a fine scissor. The human tubal fluid contained 25 mM sodium bicarbonate and 3 mg/mL BSA (pH 7.2–7.4).

### *In vitro* fertilization

Superovulation and *in vitro* fertilization were performed [29]. Briefly, oocytes were recovered from superovulated female mice 13–17 h after a 7.5 U hCG (human chorionic gonadotropin) injection, and the cells were pretreated with 7.5 U of PMSG (pregnant mare's serum gonadotropin) for 48 h. Sperm were collected from the cauda epididymides of WT and MTMR14^-/-^ mice and capacitated in human tubal fluid at 37°C for 1 h. Capacitated sperm suspension was added to the fertilization droplets to yield a motile sperm concentration of 1.5–2*10^6^/mL. After six hours of co-incubation, the oocytes were moved into M2 medium. The presence of double pronuclei was considered fertilization at 10–11 h after fertilization.

### Ratio of testis/body weight

WT and MTMR14^-^/^-^ mice at six and twelve weeks were weighed and sacrificed to isolate their testes. Then, the weights of these testes were also determined. The ratios of testis weight to body weight were calculated.

### Histological analysis

Histological analysis of testes from WT and MTMR14^-^/^-^ mice was performed as previously described [30]. Briefly, testes fixed in Bouin’s solution and embedded in paraffin were cut into 3 μm sections using a Leica SM2000R microtome (Leica, Wetzlar, Germany). These sections were stained with haematoxylin and eosin for optical microscopy.

### Sperm counts

Mature sperm from WT and MTMR14^-^/^-^ mice were isolated as described above. The sperm were transferred into 3 mL of DMEM and incubated at 37°C/5% CO_2_ for 30 min. After capacitation, the sperm from WT and MTMR14-/- mice were divided into two aliquots. One aliquot was then taken and fixed in 4% PFA to count the total number of sperm, while the other aliquot was used to count the number of non-motile sperm, which was subtracted from the total number of spermatogonia to obtain the number of motile sperm.

### Morphological assessment of spermatozoa

Mature sperm from WT and MTMR14^-^/^-^ mice were isolated as described above. The sperm were collected in a micro-centrifuge tube and kept on ice for 30 min. The tubes were centrifuged at 600 × g for 5 min to pellet the sperm. Then, the pellets were re-suspended in PBS and kept on ice to inhibit sperm motility until use.

For fixation, the sperm pellets were resuspended in freshly prepared 4% PFA in PBS and incubated on ice for 1 h. The fixed sperm was mounted on clean slides and sealed with coverslips. Sperm morphology was analysed with an Olympus microscope (Melville, NY, U.S.A.) using differential interference contrast optics. From each slide, randomly selected fields were observed, and the proportions of defective sperm were counted. Sperm with the following morphological characteristics were counted as defective: malformed heads, hairpin bends at the middle or tail of the sperm.

### Electron microscopy

For transmission electron microscopy, cauda epididymides from age-matched WT and MTMR14^-/-^ male mice were placed immediately into fixative solution containing 3% glutaraldehyde, prepared as described [31]. Selected areas were sectioned and examined using a Hitachi H-600 electron microscope.

### Analysis of sperm acrosome reaction

For the acrosome reaction, spermatozoa were capacitated for 1.5 h in Tyrode’s medium supplemented with 3 mg/mL fatty acid-free BSA and 25 mM sodium bicarbonate and then incubated for 5–20 min at 37°C in 5% CO_2_ with Tyrode’s medium plus 20 μM calcium ionophore A23187 (Sigma-Aldrich). For the determination of the proportion of sperm that had undergone an acrosome reaction, sperm were fixed and stained with Coomassie brilliant blue R250 as described previously [25]. At least 200 spermatozoa from each male were examined for the presence or absence of the characteristic dark blue crescent.

### TUNEL

TUNEL assay for apoptotic cell detection was performed using the *In Situ* Cell Death Detection Kit (Boehringer Mannheim GmbH, Mannheim, Germany) according to the standard protocol. Testis tissues from 4 pairs of WT and MTMR14^-/-^ mice were used for TUNEL analysis. The TUNEL-positive spermatogenic cells in approximately 100 seminiferous tubules of each mouse were counted, and the apoptotic indices were then determined by calculating the ratio of total numbers of TUNEL-positive cells to the numbers of counted seminiferous tubules. The software used for the statistical analysis was Image Pro-Plus, version 6.0.

### Apoptosis detection by flow cytometry

Sperm from WT and MTMR14^-/-^ mice were freshly collected and washed with PBS twice. The detection of apoptosis by flow cytometry was performed according to the instruction manual of the FITC-Annexin V apoptosis detection kit (BD Biosciences, San Diego, CA, USA). Briefly, 5*10^5^ cells were resuspended in 500 μL of Annexin V binding buffer, and 5 μL of Annexin V-FITC and 5 μL of PI were added. The cells were gently mixed and incubated for 10–15 min in the dark. Within one hour, the cells were analysed by flow cytometry (Coulter Epics XL, Beckman). The data were analysed using WinMDI software.

### Tension measurements of mouse vas deferens

Muscle contraction of mouse vas deferens was measured as described elsewhere with some modifications [32,33]. Briefly, following cervical dislocation, vas deferens from age-matched WT and MTMR14^-/-^ male mice weighing 20–25 g were isolated. Vas deferens were suspended in individual organ baths containing oxygenated physiological saline solution [PSS, containing (in mM) 135 NaCl, 5 KCl, 1 MgCl_2_, 2 CaCl_2_, 10 HEPES, and 10 glucose, pH 7.4] at 37°C and were connected to an isometric force transducer under a resting tension of 0.5 g. After 60 min of equilibration, the muscles were precontracted with high K^+^ (140 mM). Ryanodine (30 μM) was also used in this experiment to examine the inhibitory effects of the calcium channel of ER.

### Calcium imaging

The analysis of [Ca^2+^]_i_ in spermatozoa or mature sperm from WT and age-matched MTMR14^-^/^-^ mice was performed as previously described [34]. Briefly, the spermatozoa or mature sperm were isolated as described above and washed twice with HS buffer ((mM): 30 HEPES, 135 NaCl, 5 KCl, 2 CaCl_2_, 1 MgSO4, 10 glucose, 10 lactic acid, and 1 pyruvic acid, pH 7.4). Cells were resuspended in 1 mL of HS and incubated with 20 μM Fura-2AM for 30 min. After two washes with HS, the adherent cells were resuspended in HS and incubated for 30 min to allow for de-esterification of the dye. Fluorimetric determination of calcium concentration was performed using a Polychrome V monochromator (Till-Photonics, Gräfelfing, Germany) and/or a charge-coupled device camera coupled to an inverted microscope (IX71, Olympus, Germany). Intracellular calcium concentrations were determined as the proportions of detected fluorescence intensities at 340 nm to 380 nm. Fluorescence images were acquired and analysed with the Metafluor for Olympus software.

### Statistics

The results are expressed as the mean ± SD. Student’s t-test was performed using Origin software, version 9.0 (OriginLab, Northampton, USA). *P* < 0.05 was considered statistically significant.

## Results

### Expression of MTMR14 in murine testis, spermatogonia and mature sperm

**Fig 1:**
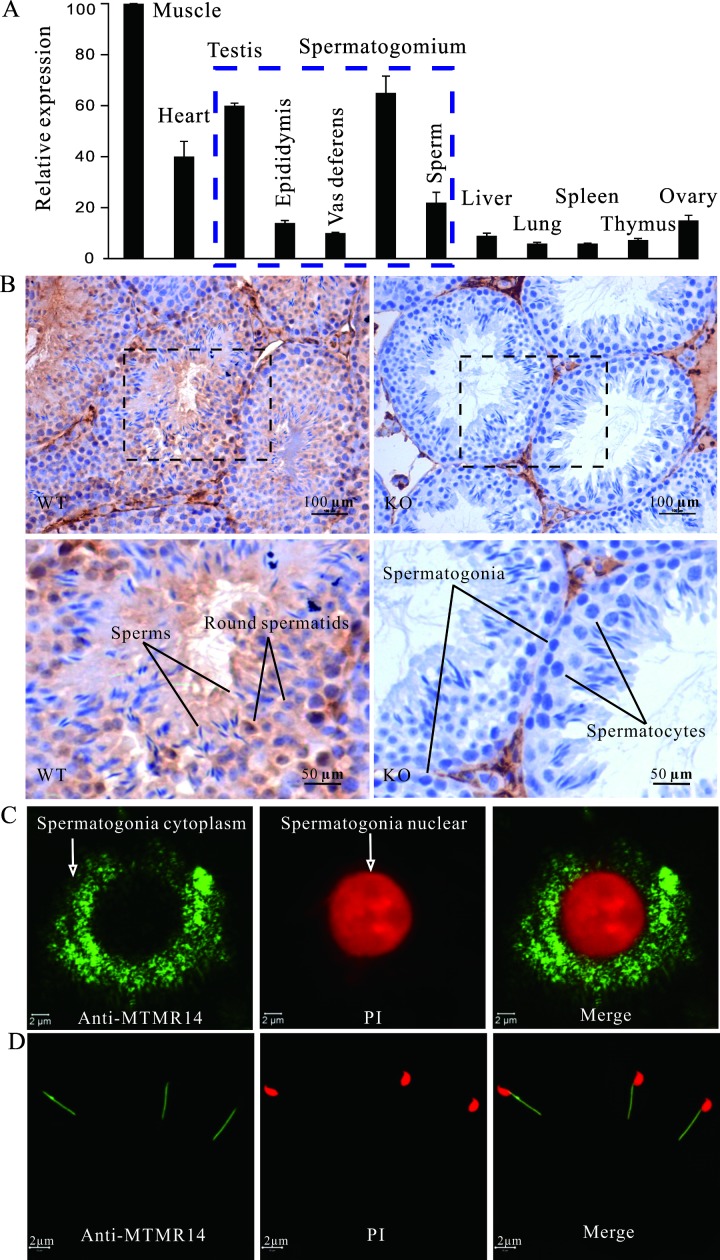
MTMR14 was expressed in mouse testes, spermatogonia, and mature sperm. **(A)** Real-time PCR analysis of mRNA expression profiles of MTMR14 in mice. The tissues of mouse skeletal muscle, heart, testis, epididymis, vas deferens, spermatogonium, sperm, liver, lung, spleen, thymus, and ovary were harvested. Then, their total mRNA levels were prepared, reverse transcribed, and analysed by real-time PCR. Beta-actin was used as a control. (B) Immunohistochemical localization of MTMR14 in mouse testes. Testis sections were obtained from KO/WT male mice, incubated with primary anti-MTMR14 antibody, and counterstained with haematoxylin and eosin. The scale bar was 100 μm. The sections from KO mice testes were used as negative controls and did not show any labelling. (C) Immunofluorescence detection of MTMR14 in spermatogonia; single spermatogenic cells were isolated from WT mice and incubated with primary anti-MTMR14 antibody and FITC-conjugated secondary antibody. After counter-staining with PI, the fluorescence signal was detected using a confocal microscope. The scale bar was 2 μm. Separate replicate experiments in both WT and MTMR14 mice were performed at least three times. (D) Immunofluorescent detects of MTMR14 in mature mouse sperm. Mature sperm were isolated from mouse vas deferens and stained as described above. These data demonstrated that MTMR14 was expressed in mouse testis, spermatogonium, and mature sperm, suggesting that MTMR14 plays a role in male fertility.

Our previous paper reported that MTMR14 is highly expressed in heart and skeletal muscle, as determined by northern blotting and western blotting analysis [18]. However, the expression of MTMR14 at the mRNA and protein levels in murine testes remained unclear and was explored in this research for the first time. As shown in **Fig 1A**, MTMR14 mRNA is widely expressed in many tissues, including reproductive organs, in male mice. The mRNA expression levels in the testes and spermatogonia are relatively higher. The immunohistochemical results demonstrate that incubation of sections of WT testes with the MTMR14-specific antibody results in intense immunostaining in seminiferous tubules, whereas the negative control only shows very faint, unspecific staining or no signal (**Fig 1B**). Thus, these data indicate that the MTMR14 protein exists in seminiferous tubules.

To further confirm the expression of MTMR14 in the reproductive organs of male mice, we then immunostained the spermatogonia from testes or mature sperm from epididymides and vas deferens using the anti-MTMR14-specific antibody. As shown in **Fig 1C**, the spermatogonia exposed to the anti-MTMR14 antibody exhibited a fluorescein isothiocyanate (FITC)-derived fluorescence pattern in the cytoplasm but not in the nucleus. However, mature sperm showed an obvious signal in the midpiece of the sperm, not in the head or tail (**Fig 1D)**. The negative controls did not exhibit any FITC fluorescence, although PI staining was still very obvious. The immunofluorescence localization of MTMR14 in reproductive cells was consistent with the RT-PCR results in **Fig 1A**. Taking these results together, we concluded that MTMR14 is also expressed in mouse testes, spermatogonia, and mature sperm.

### MTMR14-deficient male mice are subfertile

The testis is the main organ for male reproduction. During the routine MTMR14^+/-^ and MTMR14^+/-^ mating process, we did not observe any abnormalities in heterozygous male mice. To test whether deficiency in MTMR14 in the testes impairs male fertility, four mating experiments using WT and MTMR14^-^/^-^ mice were designed. Among these mice, group 3 was not used because male fertility is the main research focus. As shown in **Table 2**, in group 1, every female was pregnant and gave birth (28/28), with an average litter size of 7.5. However, in group 2, half of the WT female mice (14/28) did not become pregnant when mated with MTMR14^-^/^-^ male mice. For the mice that became pregnant, the litter size (7.1) was comparable to that in group 1. Moreover, the data that we obtained from group 3 were very similar to those from group 2, suggesting that deficiency in MTMR14 might exert no harmful effects on female fertility.

**Fig 2:**
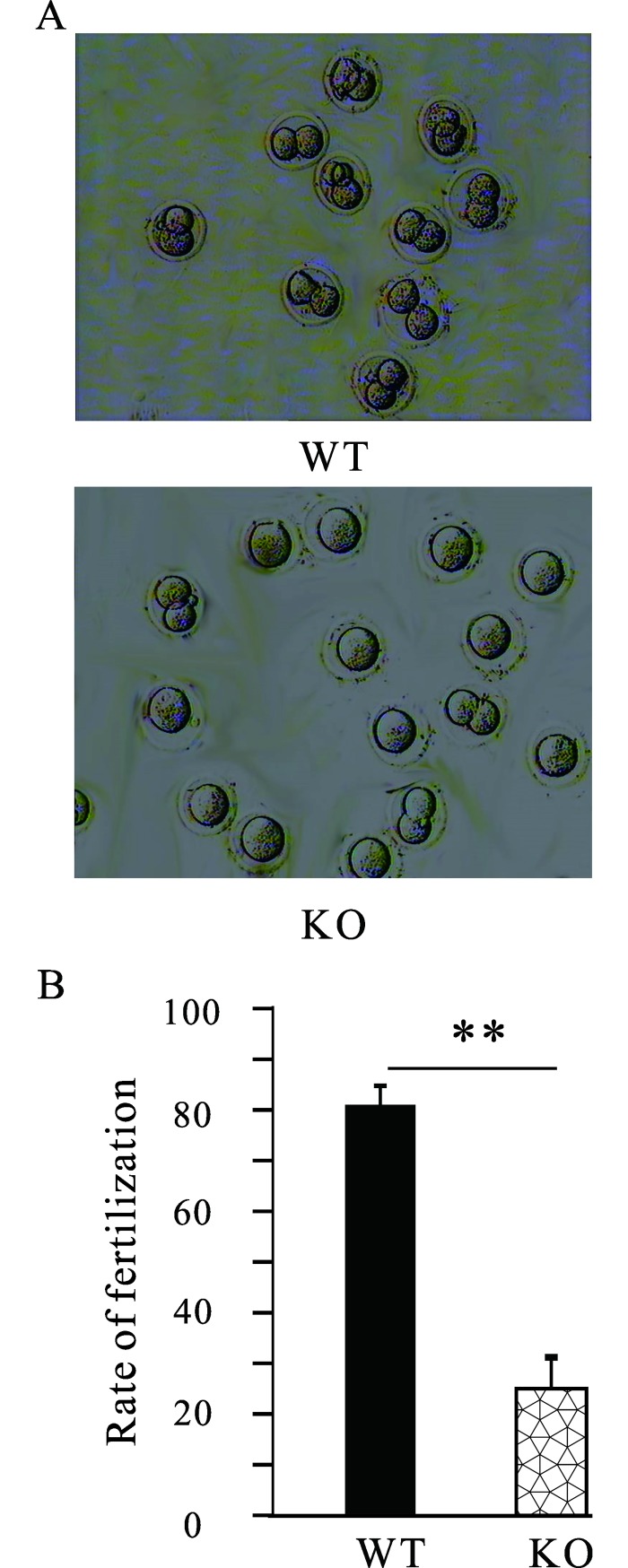
MTMR14 mutation impaired the fertilization ability of sperm *in vitro*. (A) Representative results of mouse 2-cell embryos derived from *in vitro* fertilization using sperm from WT and MTMR14^-/-^ mice, respectively (n = 4). (B) Summary of fertilization ratios of oocytes fertilized using WT and MTMR14^-/-^ mice. The proportions of fertilization for WT and MTMR14^-/-^ groups were 81.3% (130/160) and 25.3% (40/158), respectively. **: *P* < 0.01. These results indicated that MTMR14 deficiency damages fertility in male mice.

**Fig 3:**
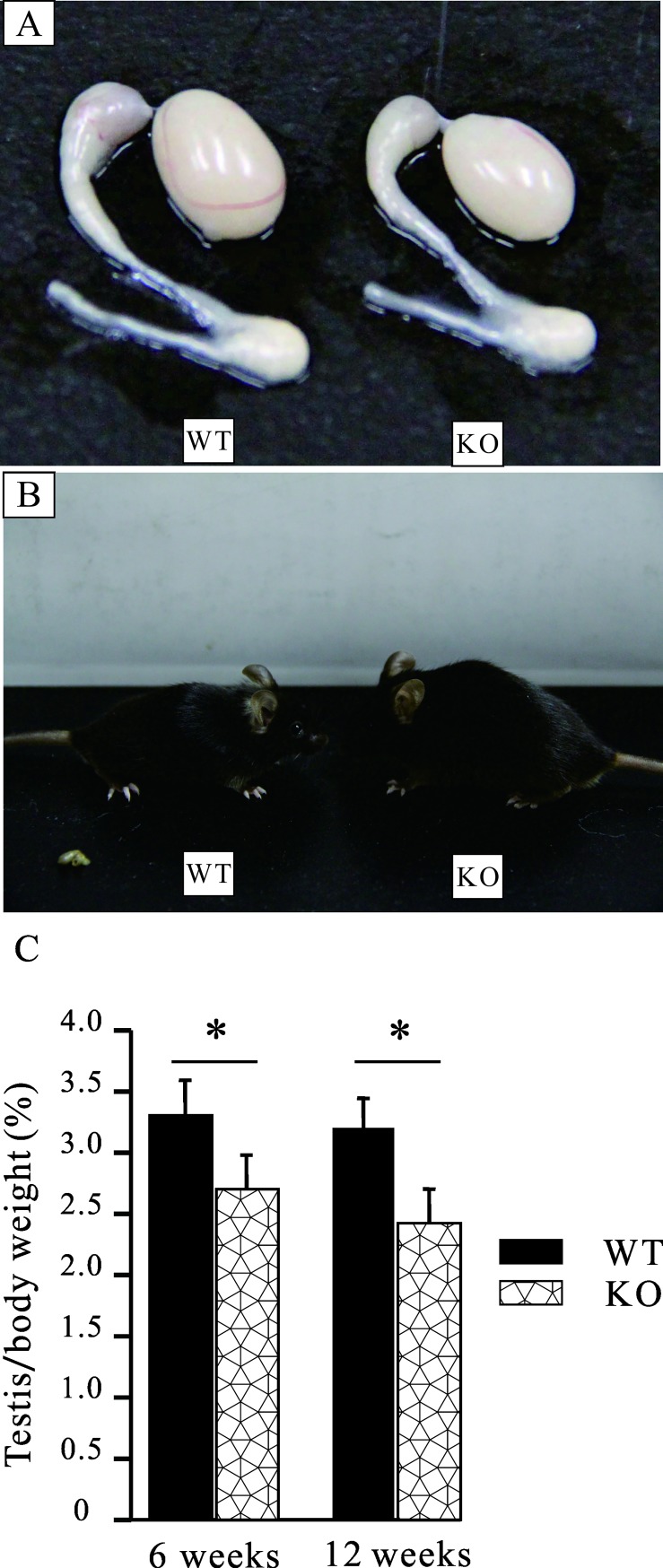
Analysis of male reproductive organs in MTMR14^-/-^ mice. (A) Representative examples of whole-mount preparations of reproductive organs from WT and MTMR14^-/-^ mice. (B) Representative photos showing that MTMR14^-/-^ mice are fatter than their littermates. (C) The ratios of testis to body weights at six and twelve weeks were significantly decreased in MTMR14^-/-^ mice (n = 10). *: *P* < 0.05. These data demonstrated that MTMR14 deficiency alters the normal size of reproductive organs in male mice, suggesting that MTMR14 participates in regulating the growth of male mice, especially the reproductive organ testis.

To further confirm the phenotype obtained above, *in vitro* fertilization was then performed. The superovulated oocytes from C57BL/6 WT mice were collected and divided into two parts randomly and then were incubated with the sperm from age-matched WT and MTMR14^-/-^ mice. As shown in **Fig 2**, *in vitro* fertilization ability of sperm from MTMR14^-/-^ mice significantly decreased. This phenotype prompted us to take a closer look at the testes of MTMR14-deficient mice. Then, WT and MTMR14^-/-^ male mice were examined regarding their overall morphological features, considering the ratio of testis weight to body weight. The organs of the testes were dissected, and images were obtained, and we found that the testes from MTMR14^-/-^ male mice were slightly smaller than those from their WT littermates, as shown in **Fig 3A**. At the same time, we also noted that the MTMR14^-/-^ mice were obviously fatter than the control mice (**Fig 3B**), resulting in a significant decrease in the ratio of testis to body weight in MTMR14^-/-^ mice. Moreover, this difference slightly increased in the older mice (**Fig 3C**). These results suggested that MTMR14 deletion could damage fertility in male mice.

### Spermatogenesis was impaired in MTMR14-deficient mice

**Fig 4:**
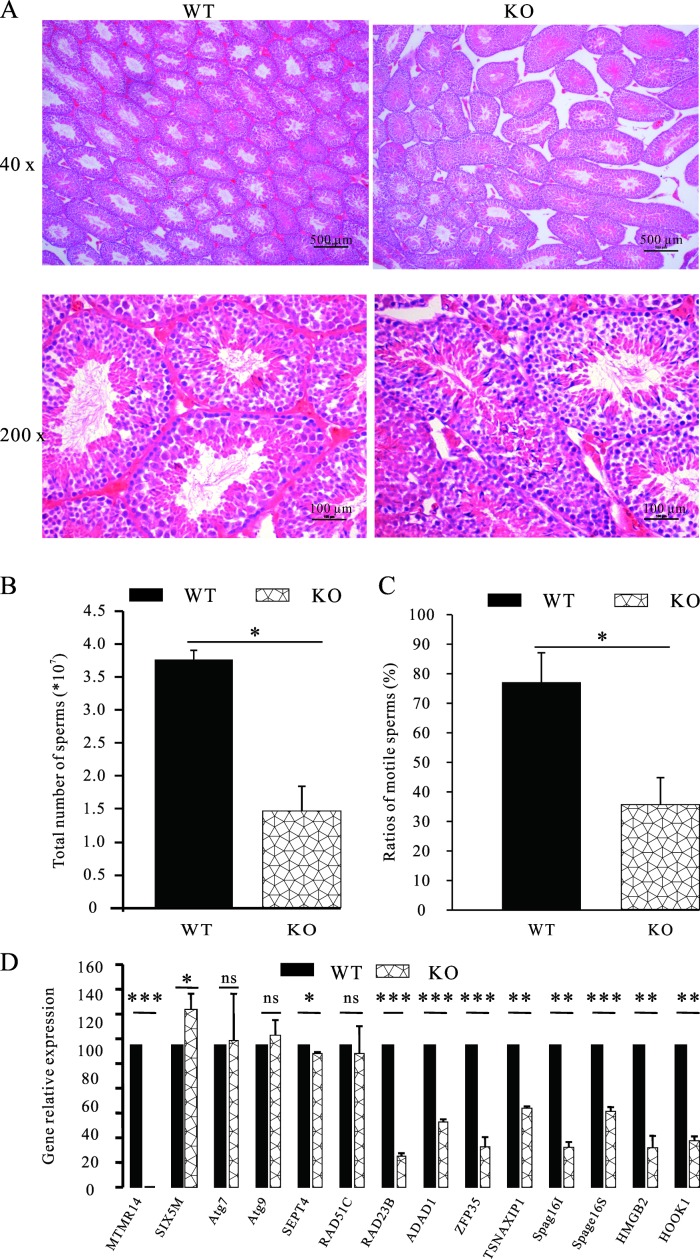
Spermatogenesis was defective in MTMR14^-/-^ mice. (A) Testicular histological sections of WT and MTMR14^-/-^ mice at eight weeks were stained using haematoxylin and eosin. The results of six separate replications were very similar. (B) Age-matched WT and MTMR14^-/-^ male mice were sacrificed to obtain the sperm from both sides of the testes, epididymis, and vas deferens. These sperm were collected and counted. The total number of sperm was reduced in MTMR14^-/-^ mice (n = 8). *: *P*: <0.05. (C) The ratio of motile sperm in MTMR14^-/-^ mice significantly decreased compared with age-matched WT mice (n = 8). *: *P* < 0.05. (D) Effects of MTMR14 deficiency on the mRNA expression levels of spermatogenic genes. RT-PCR was performed to detect the relative expression levels of the following genes: SIX5, Atg7, Atg9, SEPT4, RAD51C, RAD23B, ADAD1, ZFP35, TSNAXIP1, Spag16l, Spag16s, HMGB2, and HOOK1. MTMR14 was used as a positive control. *: *P* <0.05; **: *P* <0.01; ***: *P* < 0.001; ns: not significant. These results demonstrated that the deletion of MTMR14 damaged spermatogenesis in mice.

Testes from WT and MTMR14^-/-^ mice were dissected and examined by histological analyses. Six mice from each genotype were tested. Moreover, the proportion of motile sperm cells in MTMR14^-/-^ mice also decreased by half compared to that in WT control group (**Fig 4C**). To further confirm this finding, a series of genes that have been reported to be involved in spermatogenesis were analysed by RT-PCR. As shown in **Fig 4D**, the relative mRNA expression levels of eight genes among the thirteen genes we tested–RAD23B, ADAD1, ZFP35, TSNAXIP1, Spag16l, Spag16s, HMGB2, and HOOK1 –were all decreased in KO testes, in which MTMR14 was absent. Taken together, these data demonstrated that spermatogenesis was impaired in MTMR14^-/-^ mice.

### Structures and acrosome reactions were impaired in MTMR14^-^/^-^ sperm

**Fig 5:**
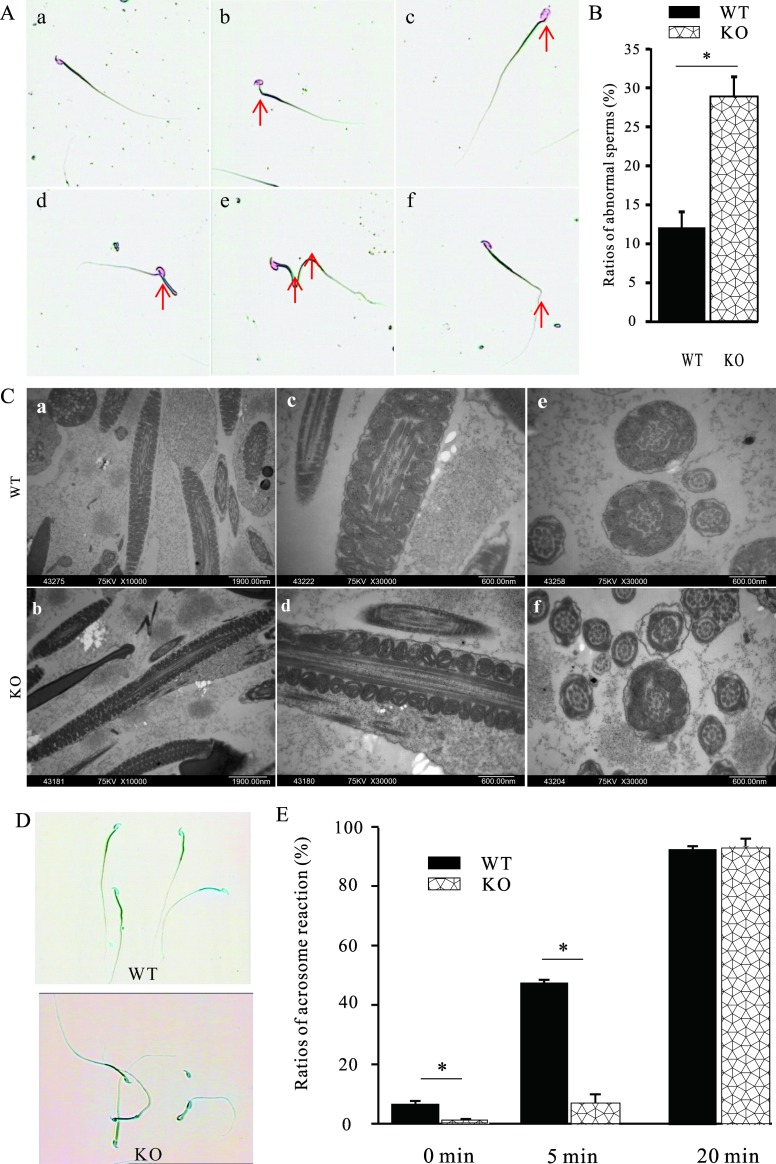
Both morphology and the acrosome reaction of sperm were impaired in MTMR14^-/-^ mice. (A) Sperm with different structures. Sperm that are normal (a) or that have abnormal junction of the head and midpiece (b), abnormal heads (c), hairpin in the midpiece (d), hairpin in the principle piece (e), and hairpin in the tail (f) structure; red arrows indicate abnormal regions. (B) Statistical data of the proportions of abnormal sperm in WT and MTMR14^-/-^ mice (n = 8). *: *P* < 0.05. (C) The ultrastructure of sperm tails in MTMR14^-/-^ mice was subtly abnormal. Representative lengthwise images of the midpiece of elongating spermatids from WT at ×10,000 (a) and ×30,000 (c) and from MTMR14^-^/^-^ mice at ×10,000 (b) and ×30,000 (d) and representative images of axonemes from epididymal sperm from WT at ×30,000 (e) and from MTMR14^-^/^-^ mice are shown. In the KO mice, the membranes of epididymal sperm tails (f) expanded and creased. (D) Representative images of sperm that underwent acrosome reactions from WT and MTMR14^**-/-**^ mice; characteristic light blue staining of the reacted acrosome is visible in WT mice, whereas in MTMR14^-/-^ sperm, the acrosome reaction was barely found. (E) Statistical data of the proportions of sperm undergoing acrosome reactions at different time points after capacitation using calcium ionophore A23187. For each sample, at least 200 sperm were checked and counted. *: *P* < 0.05. These data suggested that MTMR14 plays an important role in regulating the fertilization ability of sperm in mice.

Mature sperm from both the epididymis and vas deferens were examined under light microscopy. As shown in **Fig 5A**, there were striking differences in sperm morphology between WT and KO mice. In KO mice, approximately 28% of mutant sperm was abnormal, as reflected in abnormal heads, junctions between heads and midpieces, hairpin structures in the principal piece and in the tail, or truncated flagella, whereas only 12% of WT sperm had abnormal morphology (**Fig 5B**). At the same time, transmission electron microscopy analysis of epididymal sperm revealed that, although MTMR14^-/-^ sperm did not display any obvious loss of microtubule structure, the membranes of sperm tails expanded and creased (**Fig 5C**).

After capacitation, mature sperm must undergo the acrosome reaction before successful fusion with the oocyte. The acrosome reaction was therefore determined by modified Coomassie blue staining. Without calcium ionophore A23187, which should bypass the need to activate other calcium channels, even in WT sperm, only 5% of capacitated sperm underwent the acrosome reaction. In contrast, even fewer mutant sperm could undergo the acrosome reaction. After treatment with A23187 for only 5 min, the proportions of sperm from both the WT and the KO mice that underwent the acrosome reaction dramatically increased, with approximately 50% in WT mice but less than 10% in mutants. This finding suggests the MTMR14-deficient mice show defects in the acrosome reaction even after stimulation with A23187. However, if the treatment lasted for 20 min, almost all of sperm in both groups underwent the acrosome reaction (**Fig 5D and 5E**), while the difference between these two genotypes no longer existed. These data suggest that deficiency in MTMR14 did not cause any loss of the elements necessary for the acrosome reaction.

### Deficiency of MTMR14-induced apoptosis in testis

**Fig 6:**
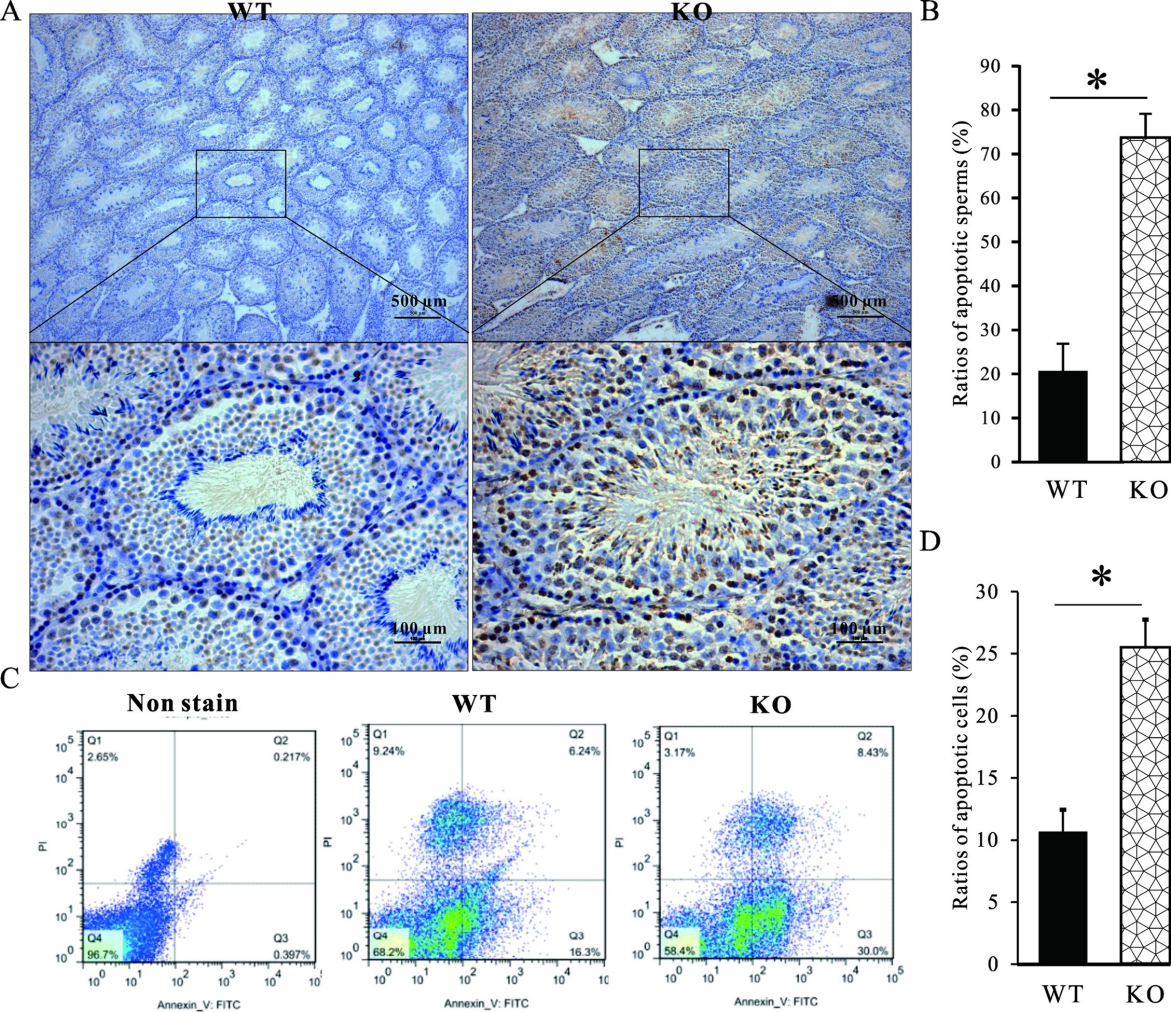
Deficiency of MTMR14 induced apoptosis and autophagy in testis. (A) Sections of testes from age-matched WT and MTMR14^-/-^ mice were used to perform the TUNEL assay. (B) The proportions of positive apoptotic cells in (A) were calculated using Image-Pro Plus software, version 6.0. *: *P* < 0.05. (C) The spermatozoa from WT and MTMR14^-/-^ littermate testes were collected and then were stained with Annexin V/PI and analysed by flow cytometry to determine the apoptotic proportions. No stained spermatozoa from WT mice were used as controls. The results of four separate experiments were very similar. Representative images of flow cytometry for the spermatozoa were shown. (D) Summary of the proportions of apoptotic cells in (C) is presented in the bar charts (n = 4). *: *P* < 0.05. These data further support that MTMR14 participates in spermatogenesis in mice.

Spermatogenesis is a highly ordered process dependent on well-balanced germ cell proliferation, differentiation and death in the testes [35]. Apoptosis is a process of programmed cell death and acts as a partner for autophagy in a cooperative manner to induce germ cell death [36]. To explain why spermatogenesis was impaired in MTMR14 KO mice, we designed the following experiments. First, TUNEL assay was performed to detect apoptosis in the sections of testes from age-matched WT and MTMR14^-/-^ mice. As shown in **Fig 6A and 6B**, we found that the proportion of apoptotic cells in MTMR14 KO testes was much higher than that in WT. To further confirm this finding, the spermatozoa from WT and MTMR14^-/-^ littermate testes were also collected and then were stained with Annexin V/PI and analysed by flow cytometry to determine the apoptotic ratio. Further, the data were in accordance with that we obtained by TUNEL assay (**Fig 6C and 6D**). All of these data suggest that deficiency in MTMR14 induced both apoptosis in mouse sperm.

### Deficiency of MTMR14 contributed to decreased muscle contraction of the vas deferens

**Fig 7:**
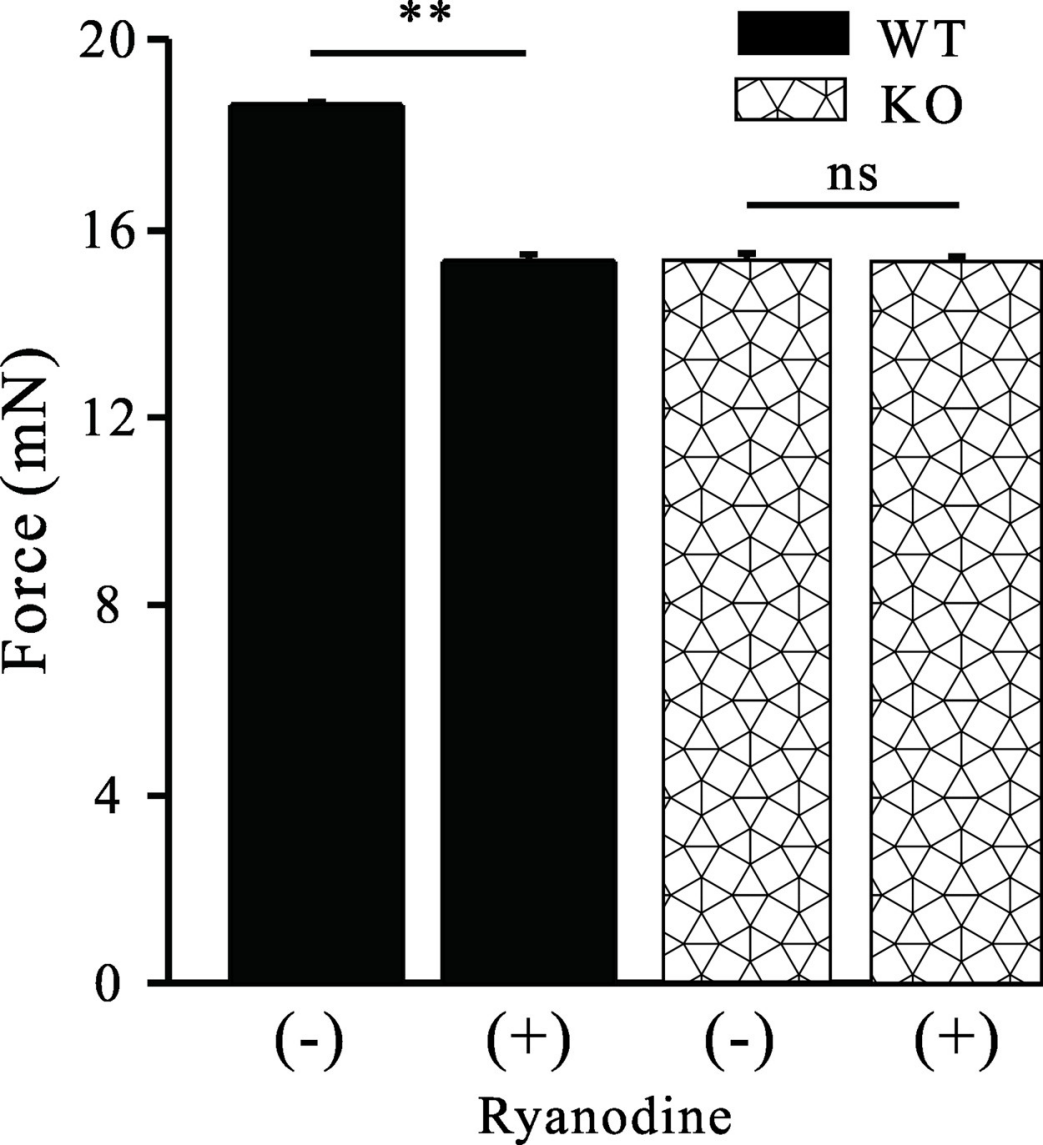
Deficiency of MTMR14 contributed to the decreased muscle contraction of the vas deferens. Vas deferens was isolated from age-matched WT and MTMR14^-/-^ mice, and muscle contractions were measured. In WT mice, ryanodine significantly decreased the contraction of the vas deferens induced by high K^+^. In contrast, in MTMR14^-/-^ mice, ryanodine failed to alter contraction of the vas deferens induced by high K^+^. **: *P* < 0.01. These data suggested that MTMR14 plays a role in contraction of the vas deferens in mice.

Contraction of the vas deferens participates in ejaculation. To uncover the mechanisms of decreased fertility in MTMR14^-/-^ mice, we evaluated high K^+^-evoked smooth muscle contractions in isolated vas deferens from age-matched WT and MTMR14^-^/^-^ mice without or with ryanodine (30 μM) treatment (n = 7). As shown in **Fig 7**, in WT mice, the contraction of the vas deferens was significantly decreased after the addition of ryanodine (*P* < 0.01), while contraction of the vas deferens in MTMR14^-^/^-^ mice was significantly less than that of WT mice (*P* < 0.01). However, contraction of the vas deferens in MTMR14^-^/^-^ mice was not affected by the addition of ryanodine (ns: no significant difference).

### Deficiency of MTMR14 impaired the homeostasis of [Ca^2+^]_i_

**Fig 8:**
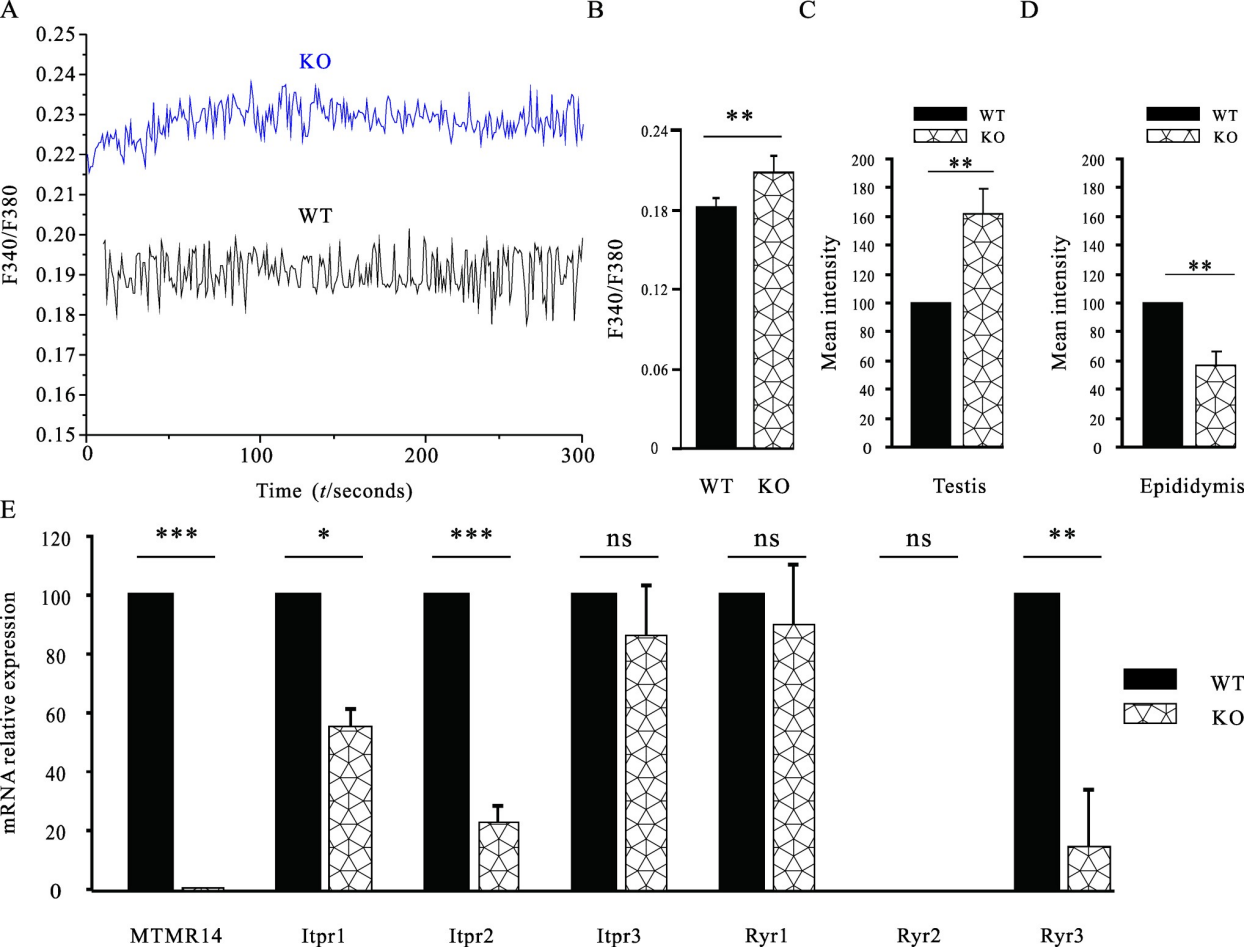
Deficiency of MTMR14 impaired the homeostasis of [Ca^2+^]_i_. (A) Basal [Ca^2+^]_i_ in WT and KO spermatogenic cells were measured by calcium imaging system (n = 8). Representative data are shown. The X axis represents time (seconds), and the Y axis represents the ratio of F340 to F380. (B) Statistical data of (A) (n = 40 cells). **: *P* < 0.01. (C) The spermatogenic cells from WT or MTMR14^-/-^ mouse testes were stained with Fura-2 AM at a final concentration of 20 μM for 30 min. The mean fluorescence intensity was monitored by flow cytometry, calculated and shown (n = 4). **: *P* < 0.01; (D) Sperm from WT and MTMR14^-/-^ mouse epididymides were isolated to evaluate the concentrations of their [Ca^2+^]_i_, which was analysed using flow cytometry. Statistical data are shown (n = 4). (E) Real-time PCR was used to detect the relative mRNA expression levels of three IP3 receptors (Itpr1, Itpr2, and Itpr3) and three ryanodine receptors (Ryr1, Ryr2, and Ryr3) in WT and MTMR14^-^/^-^ mice testes. *: *P* < 0.05, **: *P* < 0.01, ***: *P* < 0.001, ns: no statistic difference. These results demonstrated that MTMR14 regulates the homeostasis of [Ca^2+^]_i_, suggesting that MTMR14 was involved in regulating spermatogenesis and fertilization.

Ca^2+^ plays an important role in the regulation of fertilization between sperm and oocytes. Therefore, we next determined the effects of MTMR14 deficiency on the concentrations of [Ca^2+^]_i_ in spermatogonial cells and mature sperm using calcium imaging techniques and flow cytometry, respectively. As shown in **Fig 8A,** the concentration of [Ca^2+^]_i_ in spermatogonial cells derived from MTMR14^-^/^-^ mouse testes was significantly higher than that of WT mice (*P* < 0.01). Similarly, the results of flow cytometry in **Fig 8B** also supported that MTMR14 deletion resulted in an increase of [Ca^2+^]_i_ in spermatogonial cells (*P* < 0.01, n = 25 cells/5 mice). In contrast, the concentration of [Ca^2+^]_i_ in mature sperm derived from MTMR14^-^/^-^ mouse epididymis significantly decreased, compared with that of WT mice (*P* < 0.01, n = 28 cells/5 mice). To further elucidate the effects of MTMR14 deficiency on the homeostasis of [Ca^2+^]_i_, the mRNA expression levels of six calcium channel receptors–Itpr1, Itpr2, Itpr3, Ryr1, Ryr2 and Ryr3 –in mature MTMR14^-^/^-^ sperm were examined by RT-PCR [37,38]. The results in **Fig 8D** indicated that mRNA levels of Itpr1, Itpr2 and Ryr3 all significantly decreased, consistent with decreased fertility in male mice. However, the expression levels of the other three genes were not affected. These data demonstrate that MTMR14 is involved in regulating the homeostasis of [Ca^2+^]_i_ in male mouse reproductive cells.

## Discussion

In this study, we showed that MTMR14 deficiency damaged male fertility ability. In MTMR14^-/-^ mice, spermatogenesis, sperm structure and acrosome reaction ability were also damaged, while sperm apoptosis in the testes significantly increased. Moreover, the deletion of MTMR14 in male mice decreased the smooth muscle of the vas deferens and interrupted the homeostasis of [Ca^2+^]_i_ in both the spermatogonia and sperm. These changes induced by MTMR14 deficiency led to male sub-infertility.

Our previous research showed that MTMR14 was highly expressed in smooth muscle cells and heart cells [18]. This study first reported that MTMR14 mRNA was also highly expressed in several reproductive organs and tissues, such as testis, epididymis, vas deferens, spermatogonium and sperm (**Fig 1A**). This finding was further supported by the intense expression of MTMR14 proteins in spermatogonia and mature sperm. The high expression of MTMR14 suggests that male fertility is affected by MTMR14, deficiency of which can lead to male abnormal fertility or infertility. This hypothesis is further supported by the results of both *in vitro* fertilization and *in vivo* mating experiments (**Table 2**).

To test this hypothesis, we investigated the effects of MTMR14 deficiency on male fertility both *in vitro* and *in vivo*. The *in vitro* fertilization results in **Fig 2A** proved that MTMR14 deficiency significantly decreased the proportion of fertilization in mice, while the mating results were consistent with the *in vitro* fertilization results. These data strongly suggest that the fertility of MTMR14^-^/^-^ male mice was impaired. This impairment may be due to three causes: (1) damage to spermatogenesis, (2) loss of sperm-hyperactivated motility, and (3) fertilization abilities of sperm in MTMR14 KO mice. The decreased testis/body weight in MTMR14 KO mice meant fewer sperms and decreased fertility (**Fig 3**). This phenotype may be due to the functional abnormality of male reproductive system rather than systemic effects, which showed no harmful effects on other organs/systems yet. To elucidate the underlying mechanisms of decreased fertility in MTMR14 KO mice, histological analysis of testis was performed. On the one hand, the results in **Fig 4A** demonstrated that MTMR14 deletion led to abnormal structures of seminiferous tubules and fewer spermatogonia, suggesting that MTMR14 deficiency altered the structure of the testis and spermatogenesis, which were further supported by the decreased total number of sperm in MTMR14^-^/^-^ male mice. On the other hand, the decreased proportion of motile sperm in MTMR14 KO mouse sperm suggested that MTMR14 deficiency damaged the hyperactivation ability of sperm, which could contribute to damaged fertility ability. Finally, the decreased mRNA expression levels of spermatogonial genes, such as RAD51C, ADAD1, and ZFP35, also supported that spermatogenesis was abnormal in MTMR14 KO mice.

To explore the mechanisms of decreased fertility in male mice, the changes in structure and acrosome reaction ratios in MTMR14 KO sperm were studied by microscopic and staining techniques, respectively (**Fig 4**). On the one hand, MTMR14 deletion led to an increased proportion of abnormal sperm, which could result in fewer sperm ascending from the oviduct reservoir and/or reaching the oviduct ampulla, leading to subfertility [32]. However, this might not be the main reason for decreased fertility in KO mice due to the minor correlation between abnormal structure and sperm motility. On the other hand, in KO mice, damaged fertility might be due to reduced numbers of mitochondria, which supply energy for sperm swimming and hyperactivation. At the same time, MTMR14 deficiency did not alter the proportion of acrosome reactions. This outcome further proved that MTMR14 can result in subfertility rather than infertility.

Our previous study reported that MTMR14 deficiency induced autophagy in different species [24,34]. In this research, we found that MTMR14 deficiency also caused obvious apoptosis in testes (**Fig 5**). Increased apoptosis can partly explain why the total number of sperm in KO mice significantly decreased. It is also possible that increased apoptosis in spermatogonial cells and/or mature sperm contributed to subfertility in MTMR14 KO mice.

To further determine the mechanisms of MTMR14^-^/^—^induced subfertility, the contraction force changes in the vas deferens were examined. The contractility of vas deferens induced by high K^+^ in MTMR14 KO mice was significantly decreased, which could damage ejaculation and lead to poor fertility. High K^+^ induced the activation of voltage-gated L-type Ca^2+^ channels, leading to extracellular Ca^2+^ influx into the cytoplasm and increased [Ca^2+^]_i_. The significant decrease in high K^+^-induced contraction in MTMR14 KO vas deferens suggested that Ca^2+^ played a crucial role in regulating the contraction of the vas deferens in mice. However, the reduced contractility of the vas deferens in MTMR14 KO mice failed to respond to stimulus with ryanodine, an inhibitor of internal calcium release channels, while contraction of the vas deferens in their WT counterparts significantly decreased. These data suggested that abnormal contraction of the vas deferens in KO mice might be regulated by extracellular Ca^2+^ influx rather than internal Ca^2+^ release. Taking these outcomes together, we found that the reduced high K^+^-induced contraction of the vas deferens in MTMR14 KO mice was due to homeostasis of [Ca^2+^]_I_ being interrupted.

Calcium ion leads to an increase in [Ca^2+^]_i_ via two pathways: extracellular Ca^2+^ influx through voltage-gated L-type Ca^2+^ channels; and internal Ca^2+^ release from the sarcoplasmic reticulum. The basal level of [Ca^2+^]_i_ in the spermatogonia derived from MTMR14 KO testes was significantly higher than in their WT counterparts, consistent with the observation in skeletal smooth muscles [18]. In contrast, in mature sperm derived from MTMR14 KO epididymides, the concentration of [Ca^2+^]_i_ was significantly lower than that in their WT counterparts. The decreased [Ca^2+^]_i_ in mature sperm could explain why the proportion of fertilization *in vitro* and the successful rate of mating in MTMR14 KO mice were poor. Although the changes in [Ca^2+^]_i_ in male reproductive cells at different phases (immature and mature) were different, these two changes shared a common characteristic: homeostasis of [Ca^2+^]_i_ was interrupted. It is possible that in the spermatogonia, the increased [Ca^2+^]_i_ interrupted normal spermatogenesis in MTMR14 KO mice, while in mature sperm, decreased [Ca^2+^]_i_ damaged hyperactivation and fertilization. In particular, the decreased level of calcium receptor genes, such as Itpr1, Itpr2 and Ryr3, further enhanced the effects of decreased [Ca^2+^]_i_ and contributed to subfertility in MTMR14 KO mice. However, the exact roles of calcium receptor genes in MTMR14 deficiency-induced subfertility require further investigation.

In conclusion, MTMR14 deficiency led to subfertility in male mice. The deletion of MTMR14 in mice increased apoptosis, leading to abnormal structure and damaging fertility ability in male reproductive cells. All of these alterations could be correlated with interrupted [Ca^2+^]_i_. These new findings could be helpful in elucidating the mechanisms of male infertility and in developing new contraceptive drugs.

## References

[1] Evans JP, Florman HM. The state of the union: the cell biology of fertilization. Nature cell biology. 2002;4 Suppl:s57–63. http://www.ncbi.nlm.nih.gov/pubmed/12479616. 10.1038/ncb-nm-fertilityS57 . 12479616

[2] Publicover S, Harper CV, Barratt C. [Ca2+]i signalling in sperm—making the most of what you've got. Nature cell biology. 2007;9(3):235–42. http://www.ncbi.nlm.nih.gov/pubmed/17330112. 10.1038/ncb0307-235 . 17330112

[3] Wennemuth G, Babcock DF, Hille B. Calcium clearance mechanisms of mouse sperm. The Journal of general physiology. 2003;122(1):115–28. http://www.ncbi.nlm.nih.gov/pubmed/12835474. 10.1085/jgp.200308839 . 12835474 PMC2234473

[4] Darszon A, Nishigaki T, Wood C, Trevino CL, Felix R, Beltran C. Calcium channels and Ca2+ fluctuations in sperm physiology. International review of cytology. 2005;243:79–172. http://www.ncbi.nlm.nih.gov/pubmed/15797459. 10.1016/S0074-7696(05)43002-8 . 15797459

[5] Chung JJ, Navarro B, Krapivinsky G, Krapivinsky L, Clapham DE. A novel gene required for male fertility and functional CATSPER channel formation in spermatozoa. Nature communications. 2011;2:153 http://www.ncbi.nlm.nih.gov/pubmed/21224844. 10.1038/ncomms1153 . 21224844 PMC3999383

[6] Xia J, Reigada D, Mitchell CH, Ren D. CATSPER channel-mediated Ca2+ entry into mouse sperm triggers a tail-to-head propagation. Biology of reproduction. 2007;77(3):551–9. http://www.ncbi.nlm.nih.gov/pubmed/17554080. 10.1095/biolreprod.107.061358 . 17554080

[7] Lishko PV, Kirichok Y, Ren D, Navarro B, Chung JJ, Clapham DE. The control of male fertility by spermatozoan ion channels. Annual review of physiology. 2012;74:453–75. http://www.ncbi.nlm.nih.gov/pubmed/22017176. 10.1146/annurev-physiol-020911-153258 . 22017176 PMC3914660

[8] Jin JL, O'Doherty AM, Wang S, Zheng H, Sanders KM, Yan W. Catsper3 and catsper4 encode two cation channel-like proteins exclusively expressed in the testis. Biology of reproduction. 2005;73(6):1235–42. http://www.ncbi.nlm.nih.gov/pubmed/16107607. 10.1095/biolreprod.105.045468 . 16107607

[9] Qi H, Moran MM, Navarro B, Chong JA, Krapivinsky G, Krapivinsky L, et al All four CatSper ion channel proteins are required for male fertility and sperm cell hyperactivated motility. Proceedings of the National Academy of Sciences of the United States of America. 2007;104(4):1219–23. http://www.ncbi.nlm.nih.gov/pubmed/17227845. 10.1073/pnas.0610286104 . 17227845 PMC1770895

[10] Hildebrand MS, Avenarius MR, Fellous M, Zhang Y, Meyer NC, Auer J, et al Genetic male infertility and mutation of CATSPER ion channels. European journal of human genetics: EJHG. 2010;18(11):1178–84. http://www.ncbi.nlm.nih.gov/pubmed/20648059. 10.1038/ejhg.2010.108 . 20648059 PMC2987470

[11] Santi CM, Martinez-Lopez P, de la Vega-Beltran JL, Butler A, Alisio A, Darszon A, et al The SLO3 sperm-specific potassium channel plays a vital role in male fertility. FEBS letters. 2010;584(5):1041–6. http://www.ncbi.nlm.nih.gov/pubmed/20138882. 10.1016/j.febslet.2010.02.005 . 20138882 PMC2875124

[12] Zeng XH, Yang C, Kim ST, Lingle CJ, Xia XM. Deletion of the Slo3 gene abolishes alkalization-activated K+ current in mouse spermatozoa. Proceedings of the National Academy of Sciences of the United States of America. 2011;108(14):5879–84. http://www.ncbi.nlm.nih.gov/pubmed/21427226. 10.1073/pnas.1100240108 . 21427226 PMC3078394

[13] Kirichok Y, Navarro B, Clapham DE. Whole-cell patch-clamp measurements of spermatozoa reveal an alkaline-activated Ca2+ channel. Nature. 2006;439(7077):737–40. http://www.ncbi.nlm.nih.gov/pubmed/16467839. 10.1038/nature04417 . 16467839

[14] Navarro B, Miki K, Clapham DE. ATP-activated P2X2 current in mouse spermatozoa. Proceedings of the National Academy of Sciences of the United States of America. 2011;108(34):14342–7. http://www.ncbi.nlm.nih.gov/pubmed/21831833. 10.1073/pnas.1111695108 . 21831833 PMC3161588

[15] Shukla KK, Mahdi AA, Rajender S. Ion channels in sperm physiology and male fertility and infertility. Journal of andrology. 2012;33(5):777–88. http://www.ncbi.nlm.nih.gov/pubmed/22441763. 10.2164/jandrol.111.015552 . 22441763

[16] Tosch V, Rohde HM, Tronchere H, Zanoteli E, Monroy N, Kretz C, et al A novel PtdIns3P and PtdIns(3,5)P2 phosphatase with an inactivating variant in centronuclear myopathy. Human molecular genetics. 2006;15(21):3098–106. http://www.ncbi.nlm.nih.gov/pubmed/17008356. 10.1093/hmg/ddl250 . 17008356

[17] Amoasii L, Hnia K, Laporte J. Myotubularin phosphoinositide phosphatases in human diseases. Current topics in microbiology and immunology. 2012;362:209–33. http://www.ncbi.nlm.nih.gov/pubmed/23086420. 10.1007/978-94-007-5025-8_10 . 23086420

[18] Shen J, Yu WM, Brotto M, Scherman JA, Guo C, Stoddard C, et al Deficiency of MIP/MTMR14 phosphatase induces a muscle disorder by disrupting Ca(2+) homeostasis. Nature cell biology. 2009;11(6):769–76. http://www.ncbi.nlm.nih.gov/pubmed/19465920. 10.1038/ncb1884 . 19465920 PMC2693472

[19] Powers SK, Reid MB. MIP/MTMR14 and muscle aging. Aging. 2010;2(9):538 http://www.ncbi.nlm.nih.gov/pubmed/20834069. doi: 10.18632/aging.100193 . 20834069 10.18632/aging.100193 PMC2984600

[20] Romero-Suarez S, Shen J, Brotto L, Hall T, Mo C, Valdivia HH, et al Muscle-specific inositide phosphatase (MIP/MTMR14) is reduced with age and its loss accelerates skeletal muscle aging process by altering calcium homeostasis. Aging. 2010;2(8):504–13. http://www.ncbi.nlm.nih.gov/pubmed/20817957. doi: 10.18632/aging.100190 . 20817957 10.18632/aging.100190 PMC2954041

[21] Vergne I, Roberts E, Elmaoued RA, Tosch V, Delgado MA, Proikas-Cezanne T, et al Control of autophagy initiation by phosphoinositide 3-phosphatase Jumpy. The EMBO journal. 2009;28(15):2244–58. http://www.ncbi.nlm.nih.gov/pubmed/19590496. 10.1038/emboj.2009.159 . 19590496 PMC2726690

[22] Gibbs EM, Feldman EL, Dowling JJ. The role of MTMR14 in autophagy and in muscle disease. Autophagy. 2010;6(6):819–20. http://www.ncbi.nlm.nih.gov/pubmed/20595810. . 20595810 10.4161/auto.6.6.12624

[23] Hnia K, Kretz C, Amoasii L, Bohm J, Liu X, Messaddeq N, et al Primary T-tubule and autophagy defects in the phosphoinositide phosphatase Jumpy/MTMR14 knockout mice muscle. Advances in biological regulation. 2012;52(1):98–107. http://www.ncbi.nlm.nih.gov/pubmed/21930146. 10.1016/j.advenzreg.2011.09.007 . 21930146

[24] Dowling JJ, Low SE, Busta AS, Feldman EL. Zebrafish MTMR14 is required for excitation-contraction coupling, developmental motor function and the regulation of autophagy. Human molecular genetics. 2010;19(13):2668–81. http://www.ncbi.nlm.nih.gov/pubmed/20400459. 10.1093/hmg/ddq153 . 20400459 PMC2883342

[25] Yang K, Meinhardt A, Zhang B, Grzmil P, Adham IM, Hoyer-Fender S. The small heat shock protein ODF1/HSPB10 is essential for tight linkage of sperm head to tail and male fertility in mice. Molecular and cellular biology. 2012;32(1):216–25. http://www.ncbi.nlm.nih.gov/pubmed/22037768. 10.1128/MCB.06158-11 . 22037768 PMC3255718

[26] Meyer D, Voigt A, Widmayer P, Borth H, Huebner S, Breit A, et al Expression of Tas1 taste receptors in mammalian spermatozoa: functional role of Tas1r1 in regulating basal Ca(2)(+) and cAMP concentrations in spermatozoa. PloS one. 2012;7(2):e32354 http://www.ncbi.nlm.nih.gov/pubmed/22427794. 10.1371/journal.pone.0032354 . 22427794 PMC3303551

[27] Robles M, Gautier C, Mendoza L, Peugnet P, Dubois C, Dahirel M, et al Maternal Nutrition during Pregnancy Affects Testicular and Bone Development, Glucose Metabolism and Response to Overnutrition in Weaned Horses Up to Two Years. PloS one. 2017;12(1):e0169295 https://www.ncbi.nlm.nih.gov/pmc/articles/pubmed/28081146. 10.1371/journal.pone.0169295 . 28081146 PMC5231272

[28] Wennemuth G, Westenbroek RE, Xu T, Hille B, Babcock DF. CaV2.2 and CaV2.3 (N- and R-type) Ca2+ channels in depolarization-evoked entry of Ca2+ into mouse sperm. The Journal of biological chemistry. 2000;275(28):21210–7. http://www.ncbi.nlm.nih.gov/pubmed/10791962. 10.1074/jbc.M002068200 . 10791962

[29] Zhao LL, Ru YF, Liu M, Tang JN, Zheng JF, Wu B, et al Reproductive effects of cadmium on sperm function and early embryonic development in vitro. PloS one. 2017;12(11):e0186727 https://www.ncbi.nlm.nih.gov/pubmed/29095856. 10.1371/journal.pone.0186727 . 29095856 PMC5667747

[30] Tanyeri MH, Buyukokuroglu ME, Tanyeri P, Mutlu O, Akar FY, Ulak G, et al Effects of long-term treatment with haloperidol, clozapine and aripiprazole on mice isolated vas deferens. Int Urol Nephrol. 2017;49(9):1561–7. http://www.ncbi.nlm.nih.gov/pubmed/28674852. 10.1007/s11255-017-1640-9 . 28674852

[31] Sampson MJ, Decker WK, Beaudet AL, Ruitenbeek W, Armstrong D, Hicks MJ, et al Immotile sperm and infertility in mice lacking mitochondrial voltage-dependent anion channel type 3. The Journal of biological chemistry. 2001;276(42):39206–12. http://www.ncbi.nlm.nih.gov/pubmed/11507092. 10.1074/jbc.M104724200 . 11507092

[32] Ho K, Wolff CA, Suarez SS. CatSper-null mutant spermatozoa are unable to ascend beyond the oviductal reservoir. Reproduction, fertility, and development. 2009;21(2):345–50. http://www.ncbi.nlm.nih.gov/pubmed/19210926. . 19210926 10.1071/rd08183

[33] Holt JD, Watson MJ, Chang JP, O'Neill SJ, Wei K, Pendergast W, et al DPI-221 [4-((alpha-s)-alpha-((2s,5r)-2,5-dimethyl-4-(3-fluorobenzyl)-1-piperazinyl)benzyl)-N,N-diethylbenzamide]: a novel nonpeptide delta receptor agonist producing increased micturition interval in normal rats. The Journal of pharmacology and experimental therapeutics. 2005;315(2):601–8. http://www.ncbi.nlm.nih.gov/pubmed/16020629. 10.1124/jpet.105.090498 . 16020629

[34] Liu J, Lv Y, Liu QH, Qu CK, Shen J. Deficiency of MTMR14 promotes autophagy and proliferation of mouse embryonic fibroblasts. Molecular and cellular biochemistry. 2014;392(1–2):31–7. http://www.ncbi.nlm.nih.gov/pubmed/24623267. 10.1007/s11010-014-2015-5 . 24623267

[35] Print CG, Loveland KL. Germ cell suicide: new insights into apoptosis during spermatogenesis. BioEssays: news and reviews in molecular, cellular and developmental biology. 2000;22(5):423–30. http://www.ncbi.nlm.nih.gov/pubmed/10797482. 10.1002/(SICI)1521-1878(200005)22:5<423::AID-BIES4>3.0.CO;2-0 . 10797482

[36] Zhang M, Jiang M, Bi Y, Zhu H, Zhou Z, Sha J. Autophagy and apoptosis act as partners to induce germ cell death after heat stress in mice. PloS one. 2012;7(7):e41412 http://www.ncbi.nlm.nih.gov/pubmed/22848486. 10.1371/journal.pone.0041412 . 22848486 PMC3405141

[37] Darszon A, Nishigaki T, Beltran C, Trevino CL. Calcium channels in the development, maturation, and function of spermatozoa. Physiological reviews. 2011;91(4):1305–55. https://www.ncbi.nlm.nih.gov/pubmed/22013213. 10.1152/physrev.00028.2010 . 22013213

[38] Beltran C. Role of Ion Channels in the Sperm Acrosome Reaction In: Buffone MG, editor. Advances in anatomy, embryology, and cell biology. Cham: Springer International Publishing; 2016 pp. 35–69. 10.1007/978-3-319-30567-7_3 27194349

